# Mr. Frederick Pearson

**Published:** 1910-05-28

**Authors:** 


Mr. Frederick Pearson, of Boston, Lincs, who died on February 21 last, left estate of the gross value of £37,591 11s. 8d., with net personalty £36,839 12s. 4d. After making provision for his family and relatives he left the ultimate residue of his estate equally to St. Thomas's Hospital, London, the Royal National Lifeboat Institution, St. Bartholomew's Hospital, and the Lincoln County Hospital.

